# Insulin Signal Transduction is Impaired in the Type 2 Diabetic Retina

**Published:** 2020-03-25

**Authors:** Youde Jiang, Li Liu, Hainan Li, Jie-Mei Wang, Jena J. Steinle

**Affiliations:** 1 Department of Ophthalmology, Visual, and Anatomical Sciences, Wayne State University, Detroit, MI USA; 2 Department of Pharmaceutical Sciences, Wayne State University, Detroit, MI USA

**Keywords:** db/db, Retina, Insulin, TNFα, Type 2 diabetes

## Abstract

Rates of type 2 diabetes are reaching epidemic levels. Yet, the tissue specific alterations due to insulin resistance are only recently being investigated. The goal of the present study was to evaluate retinal insulin signal transduction in a common mouse model of type 2 diabetes, the db/db mouse. Retinal lysates from five month old male db/db and db/+ (control) mice were collected and processed for Western blotting or ELISA analyses for insulin receptor, insulin receptor substrate-1 (IRS-1), Akt, tumor necrosis factor alpha (TNFα) and caspase 3 levels. Data demonstrate increased TNFα and IRS-1 phosphorylation on serine 307. This led to decreased Akt phosphorylation on serine 473 and increased cleavage of caspase 3. Taken together, the data suggest dysfunctional insulin signaling in the retina of the db/db mouse. insulin.

## Introduction

With increasing rates of obesity, rates of type 2 diabetes and diabetic complications are expected to rise exponentially over the next few decades (American Diabetic Association). A key feature of type 2 diabetes is a resistance to insulin. Insulin signaling is key to a number of physiological processes, including glucose metabolism, cell growth, general gene expression, and apoptosis. Studies have focused on insulin resistance in the insulin-responsive tissues with less focus on other organs, such as the retina. Type 2 diabetes and dysfunctional insulin signaling is associated with increased rates of proliferative diabetic retinopathy and macular edema in patients. We have previously reported that diabetes-induced increases in tumor necrosis factor alpha (TNFα) can cause phosphorylation of insulin receptor substrate 1 (IRS-1) on serine 307, thus inhibiting normal insulin signal transduction in retinal endothelial cells [1]. This increase in TNFα was also associated with increased cleavage of caspase 3. We found similar findings in BBZDR/Wor type 2 diabetic rats [2,3]. However, it was not clear if these findings also occurred in type 2 diabetic mouse models.

For these studies, we used the db/db model of type 2 diabetes. We chose to use these mice as others have reported significant retinal changes. Work showed that intermittent fasting altered the gut microbiome in the db/db mice, which was associated with less retinal damage [4]. Additional studies also showed that diabetes in the db/db mice led to reduced diurnal oscillatory rhythms, which altered metabolic pathways [5]. Other groups reported increased permeability and inflammatory mediators in the retina of db/db mice, which was reduced by C1q/tumor necrosis factor (TNF) related protein 9 [6]. Ginsenoside Rg1 was shown to reduce retinal neurodegeneration in the db/db mouse through activation of IRS-1/protein kinase B(Akt)/glycogen synthase kinase 3 beta (GSK3β) levels [7]. Since it is clear that the retinas of db/db mice have damage, we wanted to ascertain whether this was due to altered insulin signal transduction.

We hypothesized that retinal lysates from db/db mice would have increased IRS-1^Ser307^ phosphorylation, leading to decreased Akt levels with increased cleavage of caspase 3.

## Methods

### Mice

Five month old male db/db (BKS.Cg-Dock7^m^+/+Lepr^db^, wildtype for Dock^7m^, homozygous for Lepr^db^) and db/+ (wildtype for Dock^7m^, wildtype for Lepr^db^, from the same colony) mice were used for these experiments. Mice were purchased from Jackson Laboratory (#000642) at 2 months age and allowed to age to 5 months at the vivarium. All animal procedures meet the Association for Research in Vision and Ophthalmology requirements and were approved by the Institutional Animal Care and Use Committee of Wayne State University and conform to NIH guidelines. Animal body weights and glucose levels are in Table 1.

### Western blotting

Whole retinal lysates were collected into lysis buffer containing protease and phosphatase inhibitors. Equal amounts of protein were placed into pre-cast trisglycine gels (Invitrogen, Carlsbad, CA), and blotted onto nitrocellulose membranes. After blocking in TBST (10 mM Tris-HCl buffer, pH 8.0, 150 mM NaCl, 0.1% Tween 20) with 5% BSA, membranes were treated with a phosphorylated insulin receptor (Tyr 1150/1151), insulin receptor, phosphorylated Akt (Ser473), total Akt, phosphorylated insulin receptor substrate 1 (Ser307), total IRS-1 (Cell Signaling Technology, Danvers, MA) TNF, (Abcam, Cambridge, MA), and beta actin (Santa Cruz Biotechnology, Santa Cruz, CA) primary antibodies overnight. The following day, membranes were incubated with secondary antibodies labeled with horseradish peroxidase. Antigen-antibody complexes were visualized using Chemiluminescence (Thermo Scientific, Pittsburgh, PA). Data was analyzed on an Azure C500 machine (Azure Biosystems, Dublin, CA). Western blot band densities were measured using Image Studio Lite software.

### ELISA

A cleaved caspase 3 ELISA (Cell Signaling Technology, Danvers, MA) was done according to manufacturer’s instructions.

### Statistics

Data were assessed for changes in db/db versus db/+ control mice. Data are presented as mean ± SEM for 5 mice. P<0.05 was accepted as significant. Data was analyzed using Prism 8.0 (GraphPad software).

## Results

### Diabetes reduces insulin receptor and Akt phosphorylation

**Figure 1:**
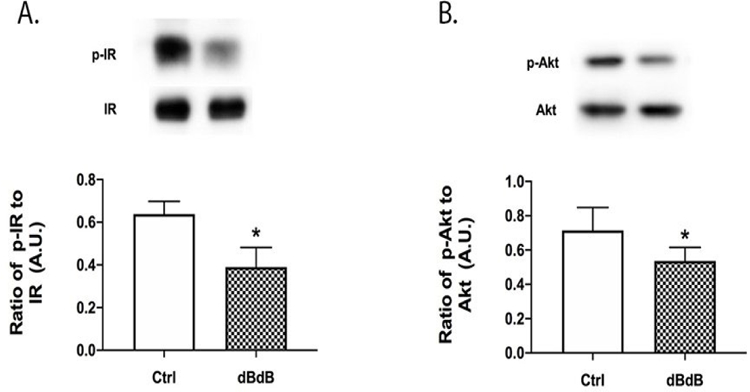
Western blotting for insulin receptor (A) and Akt phosphorylation (B) in db/+ (Control) and db/db mouse retinal lysates. *P<0.05 vs. Control. Data are mean ± SEM. N=5.

As we have shown in the retina from diabetic rats [3], diabetes significantly reduced insulin receptor and Akt phosphorylation in the db/db retina (Figure 1).

### TNFα and IRS-1^Ser307^ phosphorylation are increased in the db/db retina

**Figure 2:**
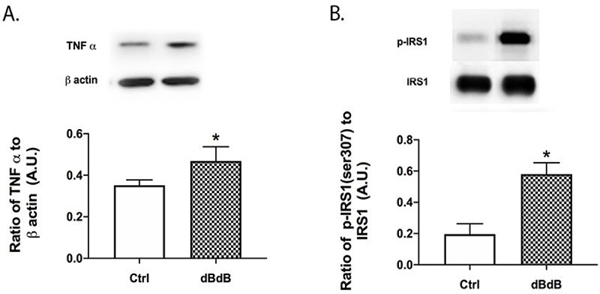
Western blotting for TNFα (A) and IRS-1Ser307 phosphorylation in db/+ (Control) and db/db mouse retinal lysates. *P<0.05 vs. Control. Data are mean ± SEM. N=5.

With Akt phosphorylation reduced in the retina of db/db mice, one possible mechanism is due to increased IRS-1^Ser307^ phosphorylation. We have previously shown that diabetes-induced increases in TNFα can increase IRS-1^Ser307^ phosphorylation in retinal cell types [1,8], which results in dysfunctional insulin signal transduction. Figure 2 shows increased TNFα and IRS-1^Ser307^ phosphorylation in the retina of db/db mice.

### Cleaved caspase 3 is increased in the type 2 diabetic retina

**Figure 3:**
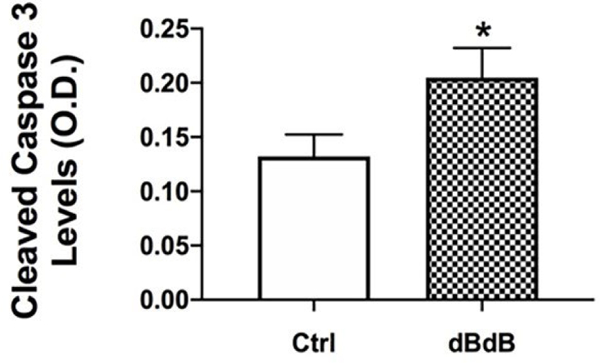
ELISA results for cleaved caspase 3 in db/+ (Control) and db/db mouse retinal lysates. *P<0.05 vs. Control. Data are mean ± SEM. N=5.

With reduced Akt phosphorylation, it follows that cleaved caspase 3 levels are significantly increased in the retina of the db/db mice (Figure 3).

## Discussion

Type 2 diabetes is characterized by insulin resistance. However, this typically applies to insulin-responsive tissues, such as muscle, adipose tissue, and the liver. We have previously reported altered insulin receptor signaling in toll-like receptor 4 knockout mice [9], miR15a mice [10], and in retinal endothelial cells [1] and Müller cells [8]. However, none of these models mimic type 2 diabetes. We have shown retina damage in the type 2 diabetic rat model, the BBZDR/Wor rat [2,3]. In this study, we wanted to investigate insulin signal transduction in the retina of db/db mice.

Studies on jejunal proteins from db/db mice showed impaired muscle insulin signaling, leading to insulin resistance [11]. Work has shown that rexinoids improved insulin signaling in skeletal muscle through decreased IRS-1^Ser307^ phosphorylation [12]. In contrast, work in hepatic tissues suggest that protein kinase C delta (PKCd) alters liver insulin signaling [13]. Focusing on the retina, studies have shown altered insulin signaling in the Streptozotocin (STZ) model of type 1 diabetes [14]. In the STZ model, PKC altered insulin receptor immunoreactivity and signaling in endothelial cells and pericytes [14]. Additionally, work in the STZ model showed that insulin receptor signaling is key to health of the retinal pigmented epithelial (RPE) cells, leading to proper photoreceptor function [15].

Our findings in the present study suggest that protein levels of key players in retinal insulin signal transduction are altered in the db/db mouse. The findings of increased TNFα levels associated with increased IRS-1^Ser307^ phosphorylation suggest that this may be the causative factor in the increased cleavage of caspase 3. It is established that the db/db mouse has altered retinal inflammation, which likely involves TNFα [6]. Increased TNFα can impair normal insulin signaling. Thus, retinal insulin signal transduction is impaired in the db/db mouse, similar to other models of diabetes.

## Figures and Tables

**Table 1:** Body weight (g) and blood glucose (mg/dl) of db/+ (Control) and db/db mice at sacrifice.

	Body weight (g)	Blood glucose (mg/dl)
Db/+	21.6	118
Db/db	>60*	>600*

* P<0.05 vs. db/+. N=5.

## References

[1] Jiang Y, Zhang Q, Soderland C, Steinle JJ. TNFα and SOCS3 regulate IRS-1 to increase retinal endothelial cell apoptosis. Cellular signalling. 2012 May 1;24(5):1086–92. 22266116 10.1016/j.cellsig.2012.01.003 PMC4073498

[2] Jiang Y, Thakran S, Bheemreddy R, Ye EA, He H, Walker RJ, Steinle JJ. Pioglitazone normalizes insulin signaling in the diabetic rat retina through reduction in tumor necrosis factor α and suppressor of cytokine signaling 3. Journal of Biological Chemistry. 2014 Sep 19;289(38):26395–405. 25086044 10.1074/jbc.M114.583880 PMC4176235

[3] Jiang Y, Thakran S, Bheemreddy R, Coppess W, Walker RJ, Steinle JJ. Sodium salicylate reduced insulin resistance in the retina of a type 2 diabetic rat model. PloS one. 2015;10(4):e0125505. 25874611 10.1371/journal.pone.0125505 PMC4397086

[4] Beli E, Yan Y, Moldovan L, Vieira CP, Gao R, Duan Y, Prasad R, Bhatwadekar A, White FA, Townsend SD, Chan L. Restructuring of the gut microbiome by intermittent fasting prevents retinopathy and prolongs survival in db/db mice. Diabetes. 2018 Sep 1;67(9):1867–79. 29712667 10.2337/db18-0158 PMC6110320

[5] Beli E, Prabakaran S, Krishnan P, Evans-Molina C, Grant MB. Loss of Diurnal Oscillatory Rhythms in Gut Microbiota Correlates with Changes in Circulating Metabolites in Type 2 Diabetic db/db Mice. Nutrients. 2019 Oct;11(10):2310. 10.3390/nu11102310 PMC6835667 31569518

[6] Li W, Ma N, Liu MX, Ye BJ, Li YJ, Hu HY, Tang YH. C1q/TNF-related protein-9 attenuates retinal inflammation and protects blood–retinal barrier in db/db mice. European Journal of Pharmacology. 2019 Jun 15;853:289–98. 30978318 10.1016/j.ejphar.2019.04.012

[7] Ying Y, Zhang YL, Ma CJ, Li MQ, Tang CY, Yang YF, Zeng JH, Huang XY, Yi J, Wang XM, He ZD. Neuroprotective Effects of Ginsenoside Rg1 against Hyperphosphorylated Tau-Induced Diabetic Retinal Neurodegeneration via Activation of IRS-1/Akt/GSK3β Signaling. Journal of Agricultural and Food Chemistry. 2019 Jul 14;67(30):8348–60. 31304751 10.1021/acs.jafc.9b02954

[8] Jiang Y, Pagadala J, Miller D, Steinle JJ. Reduced insulin receptor signaling in retinal Müller cells cultured in high glucose. Molecular Vision. 2013;19:804. 23592917 PMC3626298

[9] Liu L, Steinle JJ. Toll-like receptor 4 regulates insulin signal transduction in retinal Müller cells. Growth Factors. 2017 Nov 2;35(6):234–8. 29490521 10.1080/08977194.2018.1442833 PMC5948174

[10] Jiang Y, Liu L, Steinle JJ. miRNA15a regulates insulin signal transduction in the retinal vasculature. Cellular Signalling. 2018 Apr 1;44:28–32. 29339083 10.1016/j.cellsig.2018.01.016 PMC5884131

[11] Salinari S, Debard C, Bertuzzi A, Durand C, Zimmet P, Vidal H, Mingrone G. Jejunal proteins secreted by db/db mice or insulin-resistant humans impair the insulin signaling and determine insulin resistance. PLoS One. 2013;8(2). 10.1371/journal.pone.0056258 PMC3577828 23437106

[12] Shen Q, Cline GW, Shulman GI, Leibowitz MD, Davies PJ. Effects of rexinoids on glucose transport and insulin-mediated signaling in skeletal muscles of diabetic (db/db) mice. Journal of Biological Chemistry. 2004 May 7;279(19):19721–31. 14998989 10.1074/jbc.M311729200

[13] Zhang J, Burrington CM, Davenport SK, Johnson AK, Horsman MJ, Chowdhry S, Greene MW. PKCδ regulates hepatic triglyceride accumulation and insulin signaling in Leprdb/db mice. Biochemical and Biophysical Research Communications. 2014 Aug 8;450(4):1619–25. 25035929 10.1016/j.bbrc.2014.07.048

[14] Kida T, Oku H, Horie T, Osuka S, Fukumoto M, Ikeda T. Protein kinase C-mediated insulin receptor phosphorylation in diabetic rat retina. Graefe’s Archive for Clinical and Experimental Ophthalmology. 2019 Jul 4;257(7):1427–34. 10.1007/s00417-019-04324-z 31025213

[15] Tarchick MJ, Cutler AH, Trobenter TD, Kozlowski MR, Makowski ER, Holoman N, Shao J, Shen B, Anand-Apte B, Samuels IS. Endogenous insulin signaling in the RPE contributes to the maintenance of rod photoreceptor function in diabetes. Experimental Eye Research. 2019 Mar 1;180:63–74. 30543793 10.1016/j.exer.2018.11.020 PMC6389378

